# Glutamate Transporters EAAT4 and EAAT5 Are Expressed in Vestibular Hair Cells and Calyx Endings

**DOI:** 10.1371/journal.pone.0046261

**Published:** 2012-09-25

**Authors:** Antoine Dalet, Jérémie Bonsacquet, Sophie Gaboyard-Niay, Irina Calin-Jageman, Robstein L. Chidavaenzi, Stephanie Venteo, Gilles Desmadryl, Jay M. Goldberg, Anna Lysakowski, Christian Chabbert

**Affiliations:** 1 Pathophysiology and Therapy of Vestibular Deficits, Institute for Neurosciences of Montpellier (INSERM U1051), Montpellier, France; 2 Department of Biology, Dominican University, River Forest, Illinois, United States of America; 3 Department of Anatomy and Cell Biology, University of Illinois at Chicago, Chicago, Illinois, United States of America; 4 Department of Neurobiology, Pharmacology and Physiology, University of Chicago, Chicago, Illinois, United States of America; Texas A&M University, United States of America

## Abstract

Glutamate is the neurotransmitter released from hair cells. Its clearance from the synaptic cleft can shape neurotransmission and prevent excitotoxicity. This may be particularly important in the inner ear and in other sensory organs where there is a continually high rate of neurotransmitter release. In the case of most cochlear and type II vestibular hair cells, clearance involves the diffusion of glutamate to supporting cells, where it is taken up by EAAT1 (GLAST), a glutamate transporter. A similar mechanism cannot work in vestibular type I hair cells as the presence of calyx endings separates supporting cells from hair-cell synapses. Because of this arrangement, it has been conjectured that a glutamate transporter must be present in the type I hair cell, the calyx ending, or both. Using whole-cell patch-clamp recordings, we demonstrate that a glutamate-activated anion current, attributable to a high-affinity glutamate transporter and blocked by DL-TBOA, is expressed in type I, but not in type II hair cells. Molecular investigations reveal that EAAT4 and EAAT5, two glutamate transporters that could underlie the anion current, are expressed in both type I and type II hair cells and in calyx endings. EAAT4 has been thought to be expressed almost exclusively in the cerebellum and EAAT5 in the retina. Our results show that these two transporters have a wider distribution in mice. This is the first demonstration of the presence of transporters in hair cells and provides one of the few examples of EAATs in presynaptic elements.

## Introduction

Glutamate is a ubiquitous excitatory neurotransmitter. Control of its concentration in the synaptic cleft shapes postsynaptic currents, thus ensuring high-fidelity information transfer. This is particularly important in sensory receptors that use a continually high rate of neurotransmitter release to encode incoming stimuli. Release of neurotransmitter by retinal photoreceptors and bipolar cells, as well as by inner-ear hair cells, occurs at ribbon synapseswhich dispense glutamate in quantal packets [1]. Released neurotransmitter is cleared from the synaptic cleft by a family of integral membrane proteins, the excitatory amino acid transporters (EAATs). Five EAAT isoforms have been cloned [2]–[4]. EAAT1-2 are mainly expressed by glial cells, whereas EAAT3-5 are neuronal transporters. The glutamate transporters differ in the ways that they clear neurotransmitter. EAAT1-3 transport neurotransmitter from the extracellular space to the cell’s interior. Linked to the inward movement of each molecule of glutamate is the co-transport of 3 Na^+^ ions and a proton, together with the counter-transport of a K^+^ ion. The displacement of charges results in an inward, stoichiometric current [5], [6]. In contrast, glutamate transport *per se* is weak in EAAT4 and EAAT5 because of the slower kinetics of capture and transport, thus limiting the uptake process [4], [7]–[9]. The latter two transporters also use higher-affinity binding to regulate intercellular neurotransmitter concentration. Furthermore, a large non-stoichiometric anion current, activated by the binding of Na^+^ and glutamate, is present in EAAT4 and EAAT5 [2], [4], [8], [10]. It has been shown that this conductance can control glutamate release by hyperpolarizing the presynaptic element [11], [12]. The outward anion current in EAAT1-3 appears to be small because it is masked by the concurrent inward, stoichiometric current [13].

Two types of vestibular hair cells are present in higher vertebrates and can be recognized by differences in their afferent terminals [14], [15]. Bouton endings on type II hair cells resemble those found in the cochlea and other hair-cell organs, whereas individual calyx endings differ from other terminals in surrounding almost the entire basolateral surface of type I hair cells. Quantal neurotransmission involving AMPA receptors has been demonstrated from both kinds of hair cells [16]–[18]. NMDA receptors are also present [19] and may be functional [16]. The distinctive morphology of the calyx ending raises questions as to how glutamate, once released, is cleared from the type I synaptic cleft. In most hair-cell systems, neurotransmitter diffuses to neighboring supporting cells, where it is taken up by EAAT1 [20]. This mechanism is seemingly precluded at type I hair cells as the calyx terminal blocks the direct pathway between the synaptic cleft and supporting cells. This has led to the alternative suggestion that there is a transporter in the type I hair cell and/or the calyx ending [21], [22].

In the present study, we addressed the possibility of a hair-cell glutamate transporter. This was done by looking for a transporter-related current upon glutamate application in whole-cell recordings from vestibular hair cells. We show that an anion current, attributable to EAAT4 and EAAT5, can be recorded from type I hair cells. RT-PCR, *in situ* hybridization and immunohistochemistry provide evidence that EAAT4 and EAAT5 are expressed in vestibular hair cells. There is evidence that the two isoforms are also expressed in calyx endings.

## Materials and Methods

### Animals – Ethics Statement

Experiments were performed on young adult (3–5 week old) Swiss mice of both sexes in accordance with French Ministry of Agriculture regulations and the European Community Council Directive 86/609/EEC, OJL 358, December 18, 1986. Procedures involving animal use at the University of Illinois at Chicago (UIC) were approved by the UIC Institutional Animal Care and Use Committee. The mice were anesthetized with pentobarbital solution (1 ml/kg i.p.) or with Isoflurane, USP (Phoenix Pharmaceutical, St Joseph, MO) before decapitation. All efforts were made to minimize the number of animals used and their suffering. EAAT4-eGFP mouse [23] fixed tissue was generously supplied by Dr P. Isope (CNRS UPR 3212 5, Strasbourg, France).

### Electrophysiology

For whole-cell voltage-clamp recordings, utricles were acutely dissected and mounted in an experimental chamber containing 2 ml of artificial perilymph solution (APS) as previously detailed [24]. APS contained (in mM): 137 NaCl, 5 KCl, 2 CaCl_2_, 1 MgCl_2_•6H_2_O, 10 HEPES buffer, 11 glucose, pH 7.35 adjusted to 300 mOsm/l. The bath was continually renewed at a rate of 1 ml/min with oxygenated APS at a distance from the specimen to minimize mechanical stimulation of hair bundles. All experiments were performed at room temperature (RT, 20–23°C). Recordings were made in an experimental set up previously described [25]. Briefly, data were acquired using an Axopatch 200B amplifier (Molecular Devices, Sunnyvale, CA) controlled by a 1322-A Digidata controller (Axon Instruments/Molecular Devices Corp., Union City, CA), and the pClamp 10.2 software (Molecular Devices). They were analyzed with Origin 4.1 software (Microcal Software, Northampton, MA). Data were sampled at 1 kHz and filtered at half the sampling rate (eight-pole Bessel filter). Recording pipettes with impedances of 3–4 MΩ were filled with intracellular solution, the composition of which was adjusted according to the desired chloride equilibrium potential *(E_Cl_),* which was calculated from the Nernst equation. Concentrations were (in mM): 140 KCl, 5 NaCl, 10 HEPES, 10 glucose, 5 EGTA, 3 ATP, 1 GTP, for *E*_Cl_  =  −0.5 mV; 40 KCl, 100 K-gluconate, 5 NaCl, 10 HEPES, 10 glucose, 5 EGTA, 3 ATP, 1 GTP, for *E*_Cl_  =  −30.3 mV; and 140 KSCN, 5 NaCl, 10 HEPES, 10 glucose, 5 EGTA, 3 ATP, 1 GTP, for thiocyanate experiments. After gigaseal formation onto the basolateral membrane of hair cells and membrane disruption, the membrane capacitance (*C*m, 4.8±1.4 pF; *n = *11) and series resistance (*R*_S_, 5–11 MΩ) were estimated from the decay of the capacitive transient induced by a 10 mV pulse from a holding potential of −100 mV. *R*_S_ was compensated for up to 85% after cancellation of the capacitive transients. Voltage errors resulting from uncompensated *R*_S_ did not exceed 5 mV and were not corrected. No linear leakage compensation was performed, and the liquid junction potential of 2 mV was not corrected. Results are presented as mean ± SD. *n* values represent the total number of hair cells studied in each experimental condition. Glutamate and d-aspartate (1 mM and 100 µM, Sigma) and KSCN (1 mM, Sigma) were applied for 1 to 10 s in the vicinity of recorded hair cells using a homemade gravity-driven perfusion system.

### RT-PCR

RNA was extracted from mouse vestibular ganglion and vestibular end organs using the RNeasy Mini Kit (Qiagen, Valencia, CA). Reverse transcription was performed with oligo (dT) primers and a commercially available kit, RevertAid(tm) First Strand cDNA Synthesis Kit (Fermentas, Boston, MA). The resulting cDNA mix was used as template for subsequent PCR reactions (GoTAQ Master Mix, Promega, Madison, WI) with primers specific for EAAT4 (forward primer: ACCTAACCTTGTGGAGGCCT; reverse primer: CAAAGAAGTCCCTCAGGACA; product size 329 nt) or EAAT5 (forward: TGGTCAGCTTCTGTCAGTGC; reverse: TGAAGACGATGGGGTTCTTC; product size 246 nt). As a housekeeping gene control, we used beta actin (forward primer: GTGGGCCGCTCTAGGCACCAA; reverse primer: CTCTTTGATGTCACGCACGATTTC; product size 539 nt).

### DIG-labeled Riboprobe Synthesis for *in situ* Hybridization

Plasmids were extracted from EST clones (IMAGp998G0313989Q, imaGenes, Berlin, Germany for EAAT5; EMM1002-99867379, Open Biosystems, Huntsville, AL, for EAAT4). Antisense digoxigenin (DIG) probe was generated in a 20 µl reaction containing 1 µg linearized plasmid using DIG RNA labeling mix (Roche Diagnostics, Meylan, France) and T7 RNA polymerase (Promega, Madison, WI) following the manufacturer’s instructions. A DIG-labeled riboprobe was purified on a MicroSpin G50 column (GE Healthcare, Chalfont St Giles, UK).

### *In situ* Hybridization

Retina, cerebellum and vestibular end organs were dissected out in phosphate-buffered saline (PBS) and fixed in 2% paraformaldehyde (PFA) for 1 hr at RT. Specimens were progressively dehydrated in phosphate-buffered Tris-ethanol (PBT-EtOH) washes and stored at −20°C until used. *In situ* hybridizations were performed on whole-mounts (retina and vestibular end organs) and on cryostat sections of the cerebellum as previously described [26]. Whole mount samples were rehydrated in PBT-EtOH washes and treated with proteinase K (10 µg/ml in PBT). After post-fixation and pre-hybridization, they were hybridized with DIG-labeled riboprobes at 65°C overnight. After washes in hybridization buffer, samples were blocked in the presence of 2% blocking reagent (Roche Diagnostics) and 20% inactivated sheep serum. They were then incubated with anti-DIG-alkaline-phosphatase (AP)-conjugated antibody (Roche Diagnostics), washed and revealed with NBT/BCIP staining. After staining, samples were washed in PBS, embedded in 4% agarose (Invitrogen, Carlsbad, CA) in PBS and cut into 30 µm sections in ice-cold PBS, using a vibrating microtome (Microm/Thermo Fisher Scientific, Waltham MA).

### Western Blotting

The retina, cerebellum, vestibular end organs (maculae and cristae), and vestibular ganglia were dissected in cold 0.1M PBS (pH 7.4*)* and homogenized in RIPA buffer (Sigma, St. Louis, MO) containing 50 mM Tris-HCl, 150 mM sodium chloride, 1% nonyl phenoxylpolyethoxylethanol-40 (NP-40), 0.5% sodium deoxycholate, 0.1% sodium dodecyl sulfate (SDS), and protease inhibitor cocktail (Sigma, St. Louis, MO). Following homogenization, lysates were gently agitated at 4°C for 4 hrs. All samples were centrifuged for 10 min at 12,000 g and 4°C and the supernatant quantified colorimetrically (Pierce 660 protein assay) using a Nanodrop 1000 spectrophotometer (ThermoScientific, Wilmington, DE). Twenty micrograms of each lysate were mixed at a volume ratio of 1∶1 with Laemmli sample buffer (Bio-Rad, Hercules, CA) containing 5% β-mercaptoethanol solution. The mixture was heated at 90°C for 10 min, separated on 4–20% denaturing SDS-PAGE gels (180 V for 1 hr at RT), and transferred overnight at 35 V and 4°C onto PVDF membranes. Western blotting was performed with rabbit anti-EAAT4 (1∶5,000; Cat. No. Af390-1, Frontier Science Co. Ltd., Ishikari, Hokkaido, Japan, and Cat. No. SC-50403, Santa Cruz Technology, Santa Cruz, CA) and rabbit anti-EAAT5 antibodies (1∶15,000; Cat. No. BMP024, Medical and Biological Laboratories Co., Ltd. (MBL), Naka-ku, Nagoya, Japan, distributors in Woburn, MA) incubated for 1 hr at RT followed by incubation with horseradish peroxidise-conjugated secondary antibody (1∶30,000; Cat. No. AQ132P, Chemicon, Temecula, CA) and detection by chemiluminescence (ECL Plus; Amersham-Pharmacia, Piscataway, NJ). Four other EAAT5 antibodies and another EAAT4 antibody were used with similar results. They were obtained from Santa Cruz Biotechnology, Santa Cruz, CA (Cat. No. SC-18779, Lot No. D1103, goat anti-EAAT5; Cat. No. SC-50403, Lot No. B2201, rabbit anti-EAAT4), Immunosol Pty. Ltd., Everton Park, Queensland, Australia (Cat. No. IG1129, rabbit anti-EAAT5), and rabbit anti-EAAT5 (a generous gift from M. Roux), rabbit anti-EAAT5 (a generous gift from D.V. Pow).

### Immunohistochemistry

Temporal bones were fixed by immersion with 4% PFA in 0.1 M PBS (pH 7.4) for 1 hr at RT. Excised ampullary cristae and utricular maculae were embedded in agarose (4% in PBS) or gelatin (12% with 30% sucrose in PBS), and cut into 40 µm sections using a vibrating blade microtome (Vibratome series 1000, Technical Products International, St Louis, MO) in ice-cold PBS. Free-floating sections were permeabilized for 1 hr at 4°C in PBS containing 4% Triton X-100 for 1 hr at RT, then pre-blocked with 0.5% Triton X-100 and 1% fish gelatin (Sigma, St Louis, MO) in PBS for 1 hr at RT. Sections were incubated for 2 days at 4°C with rabbit polyclonal antibodies against EAAT4 (1∶200; Frontier) or EAAT5 (1∶100; MBL) and goat anti-calretinin (1∶200; Chemicon, Temecula, CA) diluted in the blocking solution. When used, the anti-GFP (1∶2000, Abcam, Cambridge, MA) and anti-phalloidin (1∶200; Invitrogen, Carlsbad, CA) antibodies were incubated for 24 hours. Specific labeling was revealed with secondary antibodies: Alexa 594-, Alexa 488- and Alexa 647-conjugated donkey anti-rabbit, anti-mouse and anti-goat, respectively, in the blocking solution (1∶400, Molecular Probes). After rinsing with PBS, sections were incubated overnight at 4°C, then mounted in Fluorsave Reagent or Mowiol (Calbiochem, France) and observed with a Zeiss 5 live duo laser scanning confocal microscope (MRI, Montpellier, France) or a Zeiss LSM 510 (UIC, Chicago). The specificity of EAAT4 and EAAT5 immunostaining was checked by Western blots, pre-absorbing with peptide antigen supplied by the manufacturer, labelling the cerebellum (EAAT4) and retina (EAAT5) as positive control tissue, and by omission of the primary antibody. Once again, the other five antibodies described above were also used and gave similar results. Availability of peptide antigens restricted preabsorption controls to the MBL and Santa Cruz antibodies.

### Immunogold Electron Microscopy

Forty micron sections of vestibular epithelia, cut with a vibratome, were stained with a pre-embedding method. Free floating sections were permeabilized with 0.5% Triton X-100 for 1 hr, then blocked in a solution consisting of 0.5% fish gelatin and 1% BSA for 1 hr. Sections were incubated in primary antibody (rabbit anti-EAAT4, 1∶50, Frontier; rabbit anti-EAAT5, 1∶50, MBL) for 48 hrs and secondary antibody (1∶50, ultrasmall 0.6 nm colloidal gold-labeled F(ab) goat anti-rabbit IgG, Electron Microscopy Sciences, Hatfield, PA) for 24 hrs. Colloidal gold staining was silver-enhanced (IntenSE M kit, Amersham Biosciences, Piscataway, NJ). Sections were dehydrated in a graded series of alcohols, and embedded in Araldite (Fluka Durcupan, Ronkonkoma, NY). Ultrathin sections were cut, stained with uranyl acetate and lead citrate and observed using a Hitachi 7100 electron microscope (CRIC, Montpellier, France) and a JEOL 1220 (RRC, UIC).

## Results

### A Transporter-mediated Anion Current in Vestibular Type I Hair Cells

**Figure 1:**
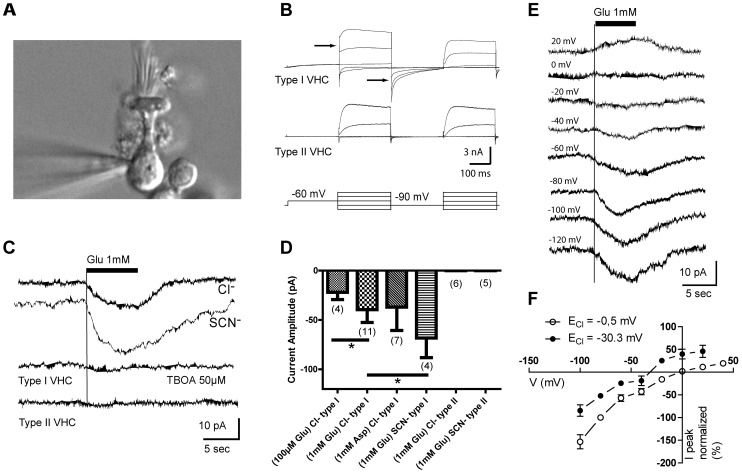
Transporter-mediated chloride conductance in partially isolated type I vestibular hair cells (*VHCs*). (**A**) Differential interference contrast micrograph of a recording electrode tip approaching the base of an amphora-shaped, type I utricular hair cell. (**B**) Electrophysiological protocol used to identify type I hair cells (*upper traces*) versus type II hair cells (*bottom traces*). As previously described [27], only type I cells exhibit *I_KL_*, an outwardly rectifying current activated at rest that was evidenced by an instantaneous current upon stepping to higher voltages (*leftmost arrow*) and deactivated by hyperpolarization *(rightmost arrow)*. (**C**) Inward current evoked in different type I VHCs voltage-clamped at −80 mV upon application of glutamate *(uppermost trace)*; inward current enhanced by substitution of thiocyanate (*SCN^–^*) for chloride *(second trace)*; the glutamate-evoked current was blocked following application of dl-TBOA *(third trace)*. Application of glutamate did not evoke a current in type II VHCs *(bottom trace)*. (**D**) Glutamate activated current under different conditions (glutamate or aspartate at various concentrations, chloride or thiocyanate anion intracellularly, and type I or type II VHCs). Each bar represents mean ± sd. Brackets with asterisk indicate *Mann-Whitney U* comparisons (*p*<0.05 for glutamate, 100 µM vs 1 mM and 1 mM Cl^-^ vs 1 mM SCN^-^); sample sizes (*n*) in parentheses. (***E***) Currents evoked by 1 mM glutamate application at various holding potentials on a type I VHC, from −120 to +20 mV in 20 mV steps. (**F**) Current-voltage relationship of glutamate-evoked responses obtained with *E*_Cl_  =  −0.5 mV (n = 7) and *E*_Cl_  =  −30.3 mV (n = 5). Peak currents at each holding potential were normalized to responses at −80 mV. Dots represent mean ± sem. When no bar is shown, the SEM was too small.

To test for the functional expression of a presynaptic glutamate transporter, we first examined the consequence of focal glutamate applications on the transmembrane currents recorded from whole-cell patch-clamped vestibular hair cells. The two hair cell types were distinguished on the basis of their morphology: amphora-shaped for type I hair cells (Fig. 1A) and cylindrically-shaped for type II hair cells [14]. The distinction was confirmed by the presence of *I_KL_*, a low-voltage activated current specific to type I hair cells [27]–[29] (see Fig. 1B and legend). When cells were held at a membrane potential of −80 mV with a Cl^–^ equilibrium potential, *E*_Cl_  =  −0.5 mV, applications of glutamate in the vicinity of the hair cells induced a slowly developing inward current in type I hair cells (Fig. 1C) with a mean peak amplitude of −39.6±13.0 pA (n = 11; range: −19 to −55 pA) for 1 mM glutamate applications and −22.0±7.4 pA (n = 4; range: −15 to −31 pA) for 100 µM glutamate applications (Fig. 1D). The glutamate transporter agonist d-aspartate (1 mM) also evoked an inward current (−37.0±23.7 pA, n = 7) (Fig. 1D). Similar currents were never evoked in type II hair cells by the application of glutamate (n = 6) or d-aspartate (n = 6) (Fig. 1C bottom trace, D). Equimolar substitution of intracellular chloride with thiocyanate (SCN^–^), a more effective carrier of the anion current, led to a significantly larger glutamate-evoked current in type I hair cells (−68.2±19.9 pA, n = 4; range: −44 to −85 pA) (Fig.1C, D), without unmasking any current in type II hair cells (n = 5). Furthermore, glutamate-evoked currents were unaffected by the presence of glutamate-receptor blockers (NMDA receptor antagonist, *dl-AP5*, 50 µM; non-NMDA receptor antagonist, *DNQX*, 50 µM; n = 12) or by the EAAT2 specific blocker, dihydrokainate (DHK) (500 µM; n = 3) [5]. Conversely, the current was reduced to 4.7±5.2% of control (n = 4) by TBOA (50 µM), a non-transportable glutamate transport blocker of all EAAT isoforms (Fig. 1C) [30]. Symmetric chloride concentrations (*E*_Cl_  =  −0.5 mV) resulted in a near-zero reversal potential (*E*_rev_) of the glutamate-evoked current, averaging −0.4±2.9 mV (n = 7) (Fig. 1E and 1F open circles). Upon changing the chloride concentration and its reversal potential *(E_Cl_*  =  −30.3 mV), there was a comparable shift in *E*_rev_ (−33.0±3.5 mV, n = 5) (Fig. 1F, solid circles). The shift of *E*_rev_ in parallel with *E_Cl_* shows that a non-stoichiometric chloride current predominates over the stoichiometric current [6], [31].

### Presence of EAAT4 and EAAT5 mRNAs in Vestibular Tissue

**Figure 2:**
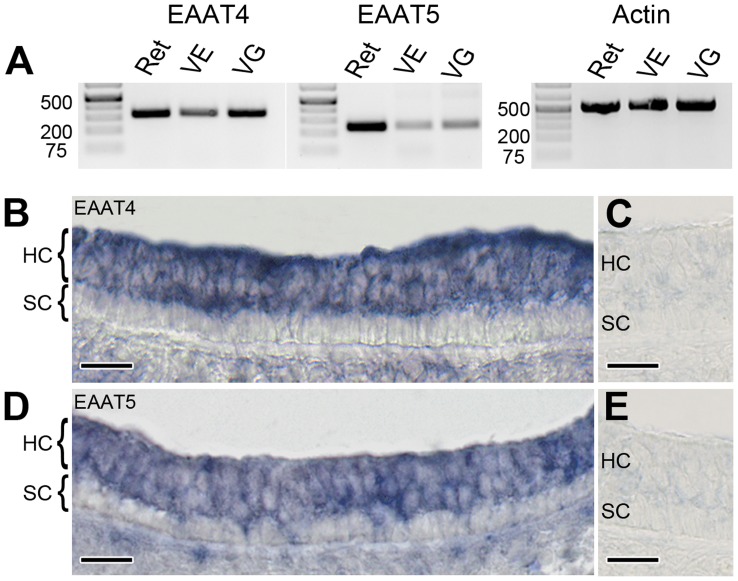
EAAT4 and EAAT5 mRNA expression in mouse tissue. (**A**) Retina (Ret), vestibular epithelia (VE), or vestibular ganglion (VG) were pooled from ten mice. RT-PCR using substrate-specific primers show expression of both EAAT4 (first panel, 329 bp) and EAAT5 (second panel, 246 bp) in various tissues. Actin PCR was used as a control (third panel, 539 bp). (**B, D**) *In situ* hybridization using EAAT4- or EAAT5-specific antisense probes in the utricular macula. Both EAAT4 and EAAT5 mRNA are found in the hair-cell layer (*HC*), but not in the supporting-cell layer (*SC*). (**C, E**) Specific sense controls showed no labeling. Scale bars, 20 µm (B–E).

The predominance of a dl-TBOA-sensitive, non-stoichiometric chloride current distinguishes EAAT4 and EAAT5 from other isoforms [2], [4]. RT-PCR was used to investigate whether the corresponding mRNAs were present in mouse vestibular epithelia and ganglia. As illustrated in Figure 2, EAAT4 and EAAT5 mRNAs were detected in both places (Fig. 2A). Primer specificity was confirmed in the retina for both transporters. *In situ* hybridization with antisense probes demonstrated that EAAT4 and EAAT5 mRNAs were restricted to the hair-cell layer (Fig. 2B, D). Although it was not possible to discriminate between type I and type II hair cells in this material, there was dense labeling of all hair cells, indicating the presence of mRNAs in both hair-cell types. Sense controls showed no specific labeling (Fig. 2C, E). Cerebellum and retina provided positive controls for EAAT4 and EAAT5 antisense probes, respectively (data not shown).

### Expression of EAAT4 and EAAT5 Proteins in Vestibular Tissue

**Figure 3:**
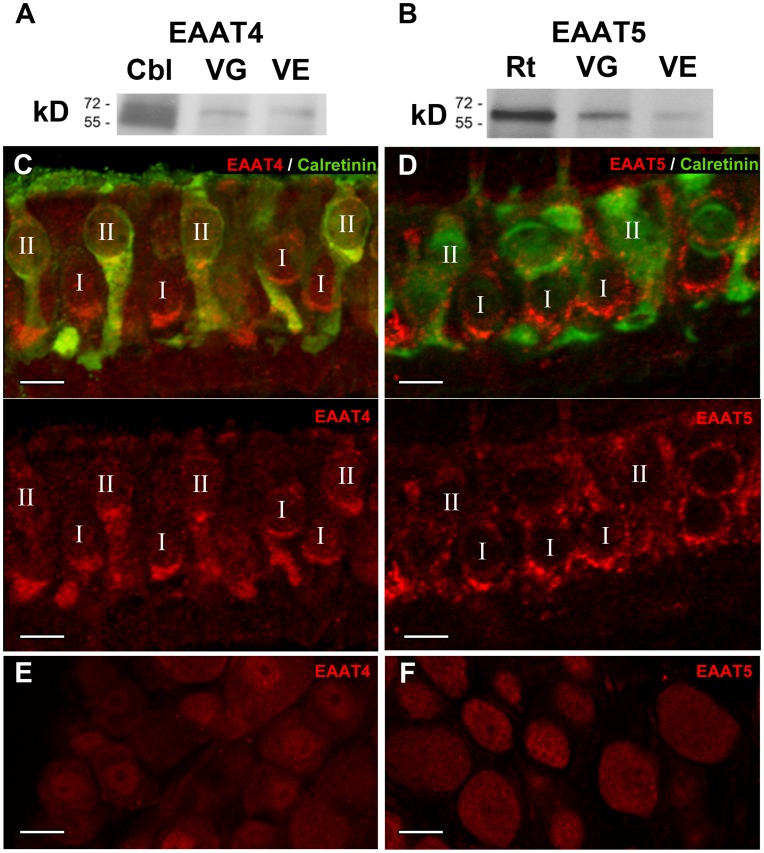
EAAT4 (*A, C, E*) and EAAT5 (*B, D, F*) protein localization in mouse tissue. Western blots using antibodies against EAAT4 (**A**, ∼61 kD) or EAAT5 (**B**, ∼62kD). EAAT4 is expressed in the cerebellum (*Cbl*), vestibular ganglia (*VG*) and vestibular organs (*VE*). EAAT5 is expressed in the retina (*Rt*), vestibular ganglia (*VG*) and vestibular organs (*VE*). Immunohistochemistry on utricular sections shows that EAAT4 (**C**) and EAAT5 (**D**) labeling (red, top and bottom panels) is conspicuous below the nuclei of both types of hair cells (*I, II*), where ribbon synapses are abundant. Calretinin labeling (green, top panels) marks type II hair cells (II) [33], [45], [46]. Immunohistochemistry of vestibular ganglion cells shows weak EAAT4 (**E**) and EAAT5 (**F**) labeling. Scale bars: 10 µm.

**Figure 4:**
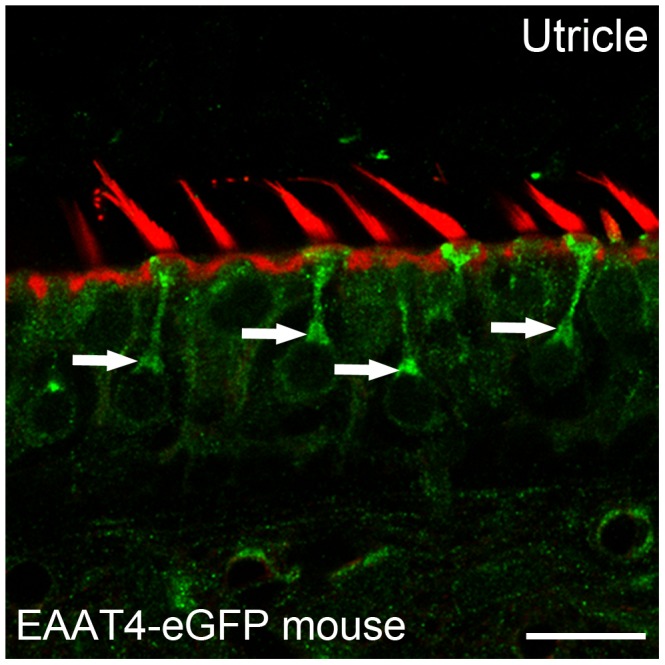
In the EAAT4-eGFP mouse, the expression of eGFP is under the control of the EAAT4 promoter[23]. The GFP fluorescence is observed in type I *(white arrows)* and type II hair cells using an anti-eGFP antibody with phalloidin red labeling of hair bundles. Scale bar: 10 µm.

**Figure 5:**
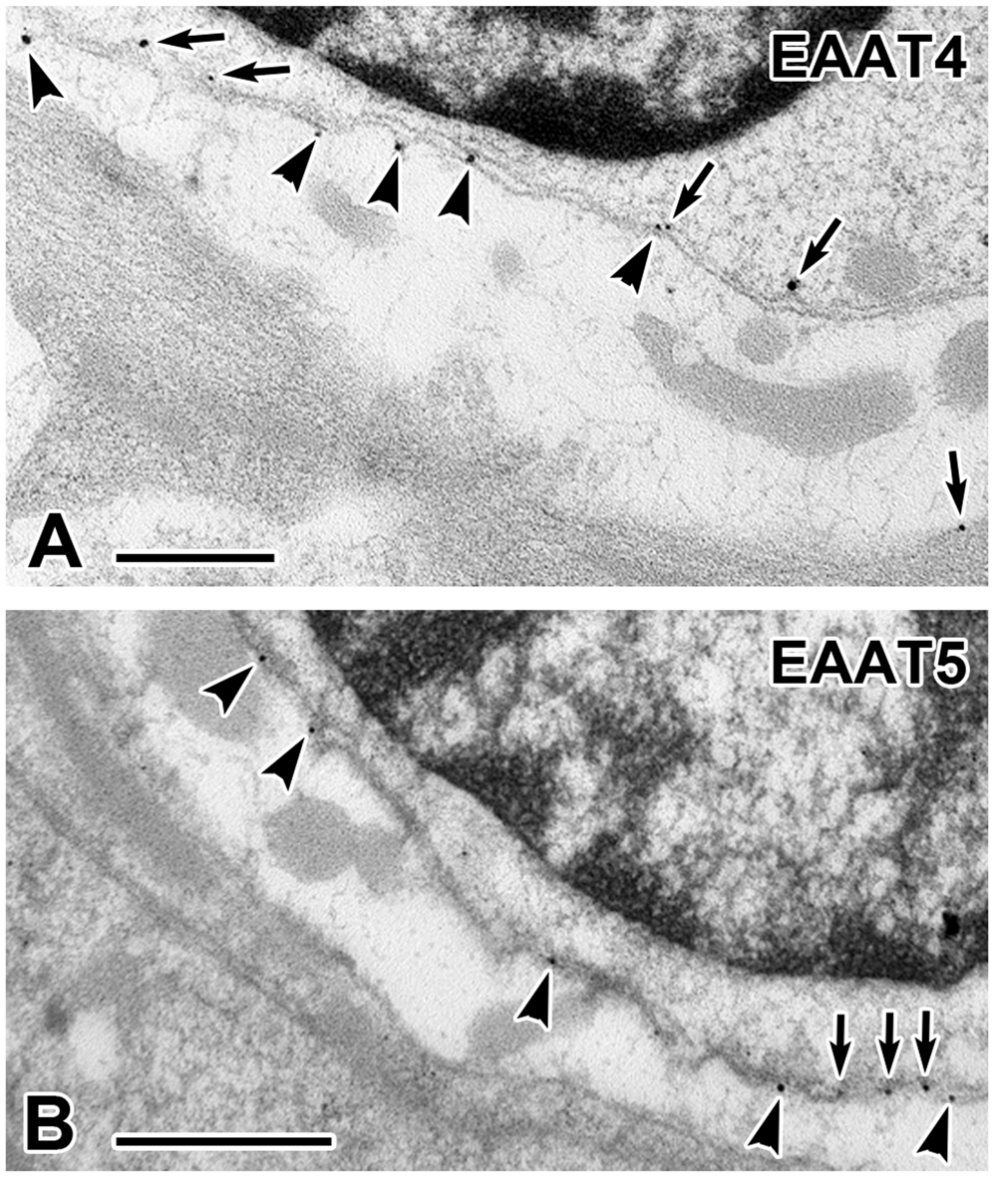
EAAT4 and EAAT5 protein localization in mouse tissue. Electron micrographs of EAAT4 (**A**) and EAAT5 (**B**) immunogold labeling particles on hair-cell membrane *(arrows),* calyx inner-face membrane *(arrowheads)* and calyx outer-face membrane *(arrow*, lower right in *A).* In both panels from top to bottom, the darkened area is a hair-cell nucleus rimmed by hair-cell cytoplasm, hair-cell and calyx inner-face membranes. The lightened area with gray mitochondria is a calyx ending whose outer-face abuts supporting cells. Scale bars: 0.5 µm.

We used Western blot analysis, confocal immunohistochemistry and immunogold electron microscopy to examine the expression of EAAT4 and EAAT5 proteins in vestibular neuroepithelia and ganglia (Figs. 3, 4 & 5). Results were similar for all five vestibular organs. Single 61 kD and 62 kD bands corresponding to the EAAT4 (Fig. 3A) and EAAT5 (Fig. 3B) polypeptides were identified in Western blots of the vestibular organs and vestibular ganglia. Similar bands were found in positive control tissues, cerebellum for EAAT4 (Fig. 3A) and retina for EAAT5 (Fig. 3B). Consistent with *in situ* hybridization results (Fig. 2B, D), confocal immunohistochemistry localized EAAT4 (Fig. 3C) and EAAT5 (Fig. 3D) to both types of hair cells, but not to supporting cells. Staining for both isoforms was heaviest below the nucleus, where ribbon synapses are found. Immunostaining for EAAT4 was similar in both hair-cell types, but that for EAAT5 was more intense in type I hair cells. Expression of EAAT4 in vestibular hair cells was confirmed in EAAT4-eGFP mice (Fig. 4) [23]. Both transporters were present in all regions of the neuroepithelia. Ganglion cells were lightly immunostained for both EAAT4 and EAAT5 (Fig. 3E, F). The cerebellum served as a positive tissue control for EAAT4 antibody and EAAT4-eGFP tissues (data not shown).

Several observations in ganglion cells supported the presence of both transporters post-synaptically, including RT-PCR (Fig. 2A), Western-blot analyses (Fig. 3A, B), and confocal immunohistochemistry (Fig. 3E, F). Because of their close proximity, the separate labeling of the calyx ending and the hair cell in the neuroepithelium could not be resolved with confocal immunohistochemistry. To eliminate the ambiguity, immunogold EM was used.

Figure 5 illustrates the distribution of immunogold particles for both transporters and Table 1 summarizes quantitative results as the density of particles per µm on the membranes of the type I hair cell and the inner and outer faces of the calyx ending. Counts were also done for the cytoplasm of the hair cell and of the ending; in the latter two structures, particles per area (A) were converted to particles per length (L); here L  =  A/Δd was the equivalent linear distance obtained as a ratio of the sampled area (A) and the distance from the membrane (Δd = 50 nm) that still qualified a 30 nm particle as being membranous. Counts were limited to a portion of the bottom of the hair cell marked by the presence of tenascin, an extracellular matrix protein associated with synapses [32], which is found in the part of the hair cell-calyx synaptic cleft populated by ribbon synapses [15], [33].

**Table 1:** Immunogold densities, EAAT antibodies, type I hair cell and calyx ending.

	EAAT4		EAAT5	
	Sample length, µm	Particle density per µm	Sample length, µm	Particle density per µm
Hair-cell membrane	45.1	0.24±0.059	62.3	0.88±0.058
Calyx inner membrane	45.1	0.22±0.060	62.3	0.91±0.29
Calyx outer membrane	64.1	0.11±0.026	51.9	0.038±0.017
Hair-cell cytoplasm	11753*	0.004±0.0005	9385*	0.002±.0007
Calyx cytoplasm	19370*	0.001±0.0003	17537*	0.001±0.0004

Sample lengths, total of five sections for each transporter. Particle density ± SE is based on a regression passing through zero between the sample length and the number of particles for each of the five samples.

* For the cytoplasm samples, length is multiplied by 0.05 µm to get the sample area.

EAAT5 particles were present with almost equal densities on the hair-cell and calyx inner-face membranes, but had negligible densities on the calyx outer face and in the two cytoplasm samples after conversion to linear densities. EAAT4 densities were only a quarter as large as those for EAAT5 on the hair-cell and inner-face membranes, were present at even lower densities on the outer face, and were negligible in the two cytoplasmic samples. Although not quantitatively examined, particles for both transporters were present on type II hair cells, but absent in supporting cells.

## Discussion

Clearing neurotransmitter from the synaptic cleft is essential in preventing receptor desensitization and excitotoxicity. In hair-cell organs, clearance is usually achieved by the diffusion of synaptically released glutamate to supporting cells, where it binds to EAAT1 and is moved intracellularly. Calyx endings, by enveloping type I hair cells, obstruct the diffusion pathway, seemingly making this mechanism unworkable. An alternative suggestion is that a glutamate transporter is present in type I hair cells, in the calyx ending or in both [21], [22].

Confirming the suggestion, we recorded a glutamate-evoked current from type I, but not type II, hair cells. As it could be nearly abolished by a transporter blocker (dl-TBOA), but not by glutamate-receptor antagonists (DNQX and AP-5), the current could be ascribed to EAAT activity. Changing the external Cl^–^ concentration shifted the current’s reversal potential to almost precisely match the equilibrium potential, E_Cl_. This last observation implied that the current was a non-stoichiometric anion current largely uncontaminated by a stoichiometric current. The results pointed to EAAT4 or EAAT5 as likely transporters, as other EAAT isoforms show more of a balance between a non-stoichiometric anion current and a stoichiometric current [34], [35]. The possibility was of general interest as EAAT4 and EAAT5 have been thought to be confined almost exclusively to the cerebellum and retina, respectively [6], [36]. Confirmation of their presence in vestibular neuroepithelia would show that one or both transporters have a wider distribution than hitherto suspected.

**Figure 6:**
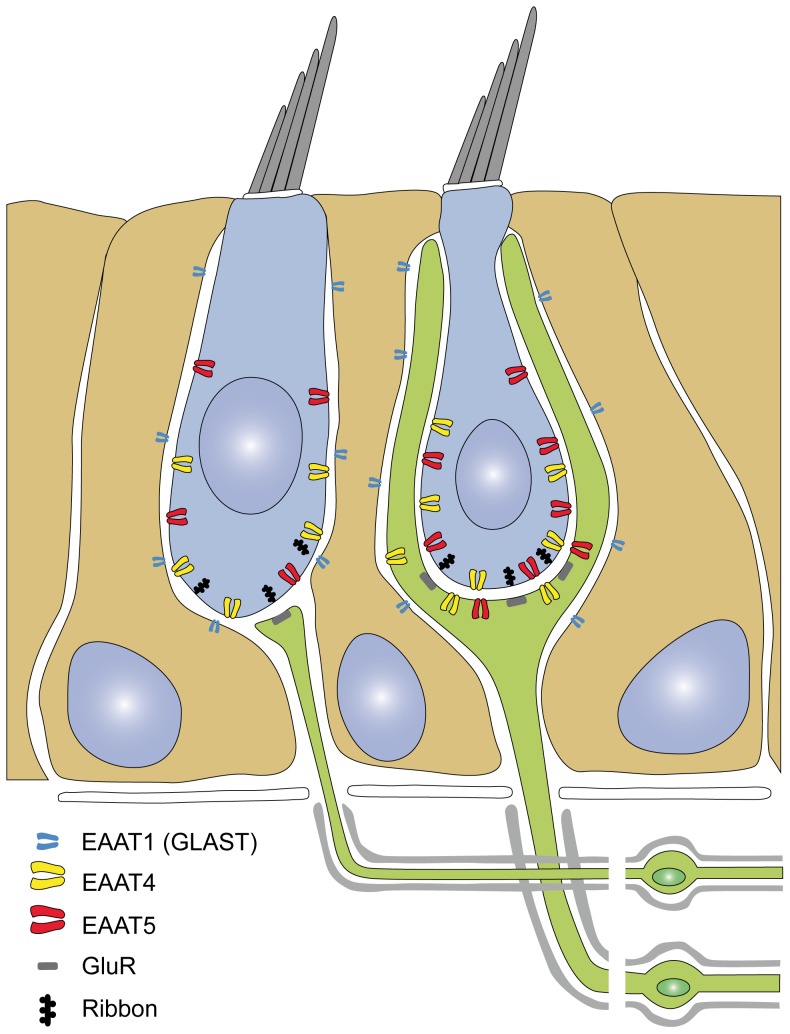
Schematic representation of EAAT4 and EAAT5 expression in the mammalian vestibular neuroepithelium. EAAT4 and EAAT5 are expressed in type I (*right*) and type II (*left*) hair cells and on the calyx inner face. EAAT4, but not EAAT5, is also expressed on the calyx outer face. EAAT5 may have a higher expression in type I versus type II hair cells. Both transporters are preferentially expressed in the subnuclear region of hair cells. EAAT1 is expressed in supporting cells [22].

Molecular and immunohistochemical methods confirmed the presence of EAAT4 and EAAT5 mRNA in hair cells and afferents. RT-PCR demonstrated the presence of mRNAs for both transporters in vestibular neuroepithelia (VE) and ganglia (VG). *In situ* hybridization confirmed that the two species of mRNAs were present in both type I and type II hair cells, but not in supporting cells. Protein expression was established in Western blots of both the VE and VG. Confocal immunohistochemistry showed that both transporters were found in type I and type II hair cells and were concentrated in subnuclear regions of the hair cells, where ribbon synapses are concentrated. Immunolabeling of ganglion cells was present, albeit weak. Hair cells and calyx endings are in such close apposition that light microscopy cannot distinguish presynaptic from postsynaptic labeling. To resolve this ambiguity, immunogold electron microscopy was used. Membrane-bound gold particles were found at both sites (Fig. 6). The specificity of postsynaptic EAAT5 labeling was indicated by its presence on the calyx inner face, but not on its outer face or on supporting cells. Some EAAT4 labeling was present on the calyx outer face, but not on supporting cells.

The suggestion that transporters would be found in hair cells and/or endings was based on the peculiar geometry of the calyx ending, which precludes a direct diffusion pathway between hair-cell release sites and supporting cells [21], [22]. A precedent for the presynaptic presence of a transporter was provided by retinal photoreceptors and bipolar cells, which use EAAT5 located presynaptically to control glutamate released to the extracellular space from ribbon synapses [4], [8], [10]. Based on these considerations, we speculated that EAAT5 would be present in type I, but not type II hair cells. While our recording of a non-stoichiometric anion current was consistent with these conjectures, including the absence of an anion current in type II hair cells, our molecular and antibody results indicate a more complicated picture, viz., that both EAAT4 and EAAT5 are present in both type I and type II hair cells and in calyx endings. It is unclear why we could not record a non-stoichiometric current in type II hair cells even when intracellular Cl^–^ was replaced by SCN^–^, a more effective charge carrier. This can be attributed to a transporter density that is too low to underlie a detectable current. It can also be hypothesized that, to become active, the transporter requires different pre-conditions in type I and type II hair cells and that only the former prerequisites were met in our experimental conditions.

### Possible Functions of EAAT4 and EAAT5

The kinetics of these two transporters differs dramatically from other members of the EAAT family. There is a strong dominance of the non-stoichiometric anion current over the stoichiometric current to such an extent that EAAT5 is considered a slow-gated glutamate receptor [6], [7], [34], [35]. The stoichiometric component is a transport current, the result of the net inward movement of two positive charges during each glutamate transport cycle. The small magnitude of this current in EAAT4 and EAAT5 is related to the low rates of glutamate transport observed for these two isoforms, as compared to EAAT1-3 [2], [4], [9], [13]. Furthermore, in addition to the intracellular translocation of glutamate at low rates, EAAT4-5 work by the high-affinity binding of the transmitter, thus buffering the extracellular glutamate concentration. In addition, when present presynaptically, the anion current can reduce neurotransmitter release by hyperpolarization or shunting inhibition [11], [12]. It has been suggested that of the two mechanisms, presynaptic inhibition might be more effective in achieving glutamate homeostasis, as high-affinity binding may have insufficient capacity to tie up more than a fraction of the glutamate release [8]. This last work was done in retinal bipolar cells. If anything, it is far from clear that high-affinity binding could serve to clear glutamate over the long distances of the intercellular cleft between the type I hair cell and the calyx ending.

As EAAT4 and EAAT5 function to regulate glutamate homeostasis in qualitatively similar ways, the question arises as to why both transporters are co-expressed where one might suffice. The presence of more than one isoform in a synaptic element is not peculiar to vestibular hair cells and calyx terminals; striking examples of co-localization are found in the retina, where all five isoforms, including splice variants, are present in photoreceptors, bipolar cells and Müller glial cells [37]. Concentrating on the present situation, there is evidence that EAAT4 has a higher affinity for glutamate with a K_M_ near 3 µM [2] compared to a K_M_ for EAAT5 near 60 µM [4]. While both transporters are adequate to prevent the continual activation of AMPA receptors, only EAAT4 would be effective at NMDA receptors with their much higher affinity for glutamate [38], [39]. Both kinds of ionotropic glutamate receptors have been immunolocalized to calyx endings [19], [40]; while synaptic activity predominantly involves AMPA receptors [16]–[18], functional NMDA receptors are also present [16]. Other sources of differentiation of the several EAAT species are their interactions with protein kinases (PKA and PKC), with glutamate transport-associated proteins (GTRAPs), and with glutamate receptors [6].

EAAT4 and EAAT5 are able to transport glutamate, but only at rates estimated to be over 50 times lower than those for the other three EAAT isoforms [4], [41]. The existence of even such low transport rates might be of importance in replenishing the glutamate store of type I hair cells. In most hair-cell systems, restoring glutamate involves the glutamate-glutamine cycle. Here, glutamate, after being released by hair cells and reaching supporting cells by diffusion, is converted to glutamine; the latter travels back to hair cells where it is converted to glutamate and re-packaged into synaptic vesicles [21]. This is a general process also used in the brain [36] and the retina [42]. But once again the presence of the calyx ending offers unique challenges by impeding the bidirectional journey between hair cells and supporting cells. Here, a hair-cell transporter could offer an alternative mechanism of glutamate recovery. Even low transport rates might suffice as the direct recovery by hair cells avoids the need for bidirectional diffusion and for the inter-conversion of glutamate to glutamine and vice versa. Possibly EAAT4 or EAAT5 could serve this purpose. An alternative is that an EAAT with substantial transporter capabilities is co-localized in the hair cell, as is the case in the retina where EAAT2 is co-localized with EAAT5 in retinal photoreceptors and bipolar cells [43], [44]. Such a transporter could also be located postsynaptically.

We have demonstrated that EAAT4 and EAAT5, previously thought to be largely confined to the cerebellum (EAAT4) and retina (EAAT5), are expressed in vestibular organs. Our findings establish vestibular hair cells, along with retinal photoreceptors and bipolar cells, as places where glutamate transporters are localized presynaptically. Present data demonstrate that the two transporters are also expressed postsynaptically. We can only speculate about their respective involvement/cooperation in glutamate homeostasis, but their co-localization offers a potentially unique opportunity to dissect their complementary roles.
